# The morphology of the pulp chamber floor of permanent mandibular first and second molars in an Indian subpopulation—a descriptive cross-sectional study employing Pawar and Singh classification

**DOI:** 10.7717/peerj.14392

**Published:** 2022-11-29

**Authors:** Ajinkya M. Pawar, Shishir Singh

**Affiliations:** 1 Department of Conservative Dentistry and Endodontics, TPCT’s Dental College and Hospital, Navi Mumbai, Maharashtra, India; 2 Department of Conservative Dentistry and Endodontics, Nair Hospital Dental College, Mumbai, Maharashtra, India

**Keywords:** Canal orifices, Mandibular molars, Morphology, Pawar and Singh classification©, Pulp chamber floor anatomy

## Abstract

**Background:**

Mandibular molars are multi-rooted teeth with a complex and strenuous root canal anatomy. Because there is relatively negligible literature describing the pulp chamber floor anatomy, predicting the exact anatomical layout and its variations is daunting. A thorough comprehension and knowledge of the same is required for efficacious endodontic treatment consequence. The purpose of this study was to characterise and ascertain the pulp chamber floor anatomy of permanent mandibular first (ManFMs) and second (ManSMs) molars in an Indian population.

**Methods:**

On 2,134 extracted human mandibular first (ManFMs = 1,067) and second molars (ManSMs = 1,067), a descriptive cross-sectional investigation was executed. Cleaning and removal of hard and soft tissue debris were performed, followed by flattening the anatomical crown until the pupal architecture was uncovered. A stereomicroscope with a magnification of 4.5 magnification was used to investigate the pulpal anatomy. Statistical analysis was carried out using chi square test, with 95% confidence intervals and a *p* value of 0.05 considered statistically significant.

**Results:**

The majority of the ManFMs showed presence of three canal orifices in 57.73%, followed by four orifices 31.31%, five orifices 8.81%, and six orifices 2.15%. For ManSMs, majorly three orifices were found in 39.83% specimens, followed by two orifices in 37.39%, four orifices in 16.31%, and single root canal orifice was observed in 6.47%. The mesio-buccal, mesio-lingual, disto-buccal, disto-lingual canal orifices exhibited round and oval shapes in both ManFMs and ManSMs. The shape was predominantly oval with a single distal canal orifice (84.25% ManFMs and 74.16% ManSMs). In ManSMs, among the single root canal orifice, 66.66% were round in shape and 33.34% were C-shaped. In ManSMs with two root canal orifices, mesially 79.44% exhibited oval and 20.56% round shape. Distally, 74.16% were oval and 25.84% were round. The shapes of the access cavities for ManFMs were rectangular in 46.67% and triangular in 53.33%. For the ManSMs, the access cavities were triangular in 39.83%, rectangular in 16.31% and non-classified (others) in 43.86% of specimen. In both ManFMs and ManSMs, the pulp chamber floor morphology was predominately Y shaped (57.73% and 39.83%) according to the Pawar and Singh classification© of pulp chamber floor anatomy.

**Conclusion:**

Our study indicated that the orifices on the pulp chamber floor are arranged in a typical manner, supporting the proposed categorization. Furthermore, description of the anatomical patterns of the pulp chamber guides the clinicians in chair-side treatment decisions.

## Introduction

Before commencing an endodontic therapy, it is critical to be conscious of the internal anatomy relationships. A thorough examination of two or more periapical radiographs is essential. These angled radiographs are quite useful in determining root canal configuration (Vertucci, 2005). In addition, clinicians should consider undertaking a 3D radiographic examination by means of cone-beam computed tomography (CBCT) promptly, especially in situations with endodontic retreatments (Abella et al., 2015; Patel et al., 2019). Knowledge of canal shape and its variations, in addition to diagnostic and treatment strategy, is a basic condition for endodontic efficiency (Estrela et al., 2014). The serious risk of overburdening the rotational endodontic tools with torsional stress and cycle fatigue, which may result in their intracanal separation, arises from performing endodontic therapy without having a thorough understanding of tooth anatomy (di Nardo et al., 2021; Zanza et al., 2021). Peters (2004) indicated that alterations in canal geometry prior to shaping and cleaning initiatives had a greater effect on the alterations that occur during preparation than the instrumentation techniques themselves, emphasising the importance of canal anatomy. It has long been documented that a root with a tapering canal and a single foreman is the quirk rather than the rule, dating back to the early work of Hess (1925).

Multiple apical foramina, extra canals, fins, deltas, intercanal connections, loops, ‘C-shaped’ canals, and auxiliary canals have been discovered by researchers. As a result, the practitioner must treat each tooth as if complicated anatomy occurs frequently enough to be deemed normal. It is additionally desirable to examine these parameters along with an assessment of the potential of implementing surgical endodontics, which is enabled with 3D diagnostic imaging, and thus to evaluate the appropriate canal characteristics in advance (Yan et al., 2021; Reda et al., 2022). The dentist must be acquainted with the various pathways root canals take to reach the apex. The pulp canal system is intricate, with canals that branch, split, and re-join. Weine classified root canal systems into four broad categories (Weine et al., 1969). Vertucci (1984) discovered a far more intricate canal system and identified eight pulp space configurations. It is also critical that the dentist has a holistic view of the root canal morphology of the tooth being treated.

The mandibular first molar (ManFM) and mandibular second molar (ManSM) are a commonly treated teeth with numerous root canal types (Maggiore, Gallottini & Resi, 1998). Variations in the morphology of the tooth pulp are influenced by genetic and environmental factors, and dentists must be made aware of the prevalence of racially determined forms (Zhang et al., 2011).

Many researchers have previously noted the anatomical intricacies of the pulp chamber, but there was no credible data on whether the position of orifices inside a single root would be indicative of the architecture of these canals (Ballester et al., 2021). To address such clinical circumstances, Pawar and Singh and propounded a new categorization in 2020, labelled the Pawar and Singh molar pulp chamber floor classification (Pawar & Singh, 2020). This classification entails describing the layout of the pulp chamber floor based on the position and numbers of canal orifices present, resulting in a unique alphabetical letter.

Thus, the purpose of this study was to assess the form of the pulp chamber floor in human permanent ManFMs and ManSMs molars from the an Indian population, thereby assessing the existence of canals, the morphology of the canal orifices, and anatomy of the pulp chamber floor.

## Materials and Methods

Ours was a descriptive cross-sectional study using an *in-vitro* research design. The study was approved by the Institutional Ethics committee of Terna Dental College (No. TDC/IRB-EC/161/2017; date of approval 09/11/2017), which waived the informed consent. This investigation was conducted on freshly extracted permanent ManFMs and ManSMs collected from various dental facilities. Since there did not exist a comprehensive evaluation of the parameters before the investigation, a stratified random sampling procedure was used to gather a total of 2,134 extracted teeth (1,067 ManFMs and 1,067 ManSMs). The Checklist for Reporting *In-vitro* Studies (CRIS) guidelines was used as the standard of methodology in the conduction of this study (Krithikadatta, Datta & Gopikrishna, 2014).

The inclusion criteria for selection were extracted permanent ManFMs and ManSMs from patients of indigenous Indian ancestry of mixed races and ethnicities (Andamanse, Austro-Asiatic, Dravidian, Indo-European, Tibeto-Burman), with fully grown and full formed roots with apices and undamaged crowns. Those teeth fractured during extraction, with previous endodontic therapy, with severely carious crowns with root caries and root canal calcifications, were excluded from current investigation.

An ultra-sonic scaler was used to clean the hard and soft tissue debris on the collected samples. These samples were immersed in 10% formalin for 5 days for disinfection (Western & Dicksit, 2016) and then were stored in a phosphate buffered solution for not more than 20 days and they were prepared for evaluation. Using a diamond disc with continuous water spray, the flattening of the crowns was done till the pulpal anatomy was visualized clearly. The pulpal anatomical structure was preserved according to the objective of our study. The sample observations were initially photographed and then recorded using stereomicroscope at 4.5× magnification. The selection of the samples considering the inclusion and exclusion criteria, sectioning the samples, and evaluation was done by the primary investigator (A.M.P.).

### Statistical analysis

**Figure 1:**
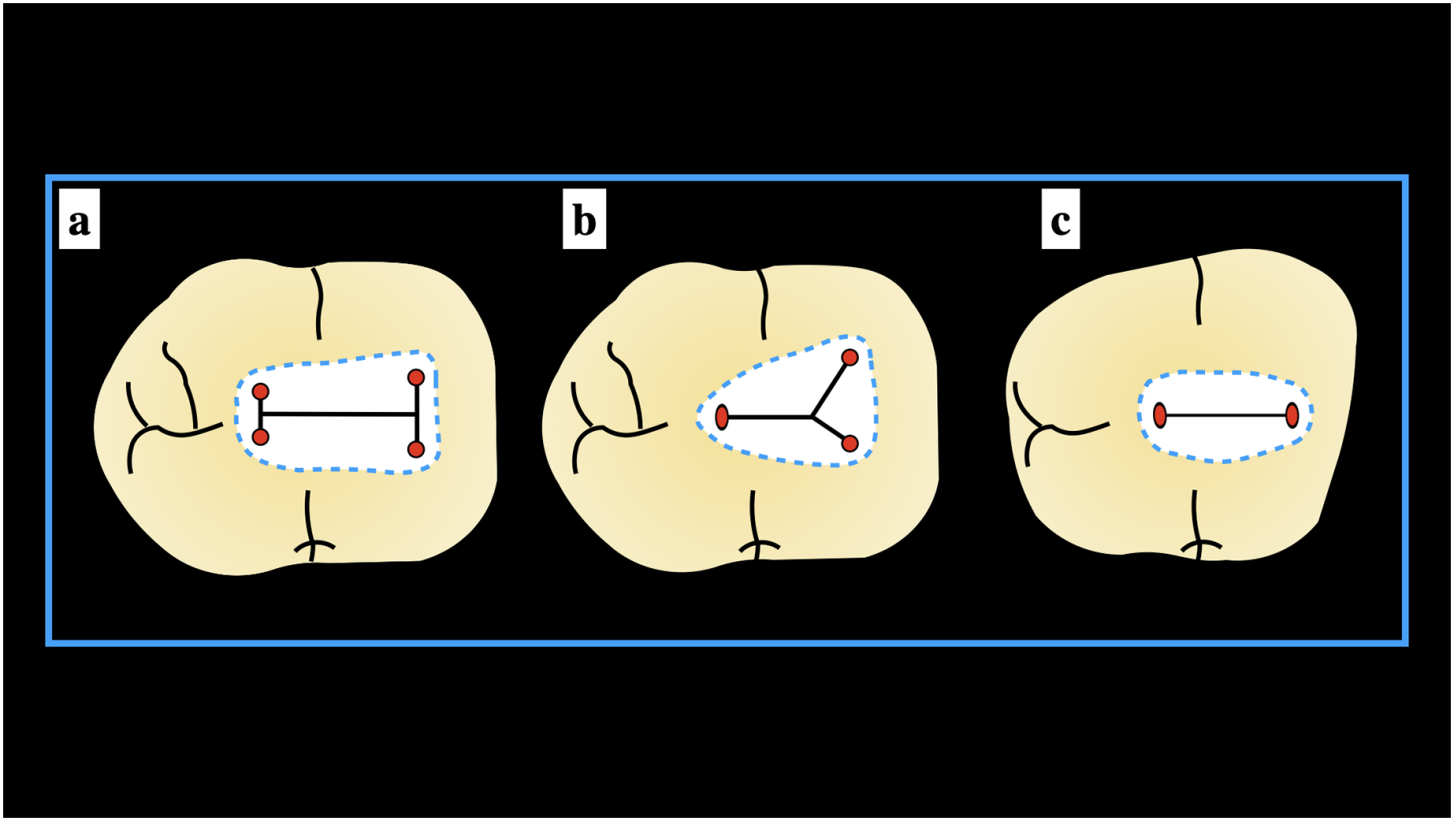
Schematic presentation of the Pawar and Singh classification© for mandibular molars (A) “H” type, (B) “Y” type, and (C) “I” type.

Data sorting was done and further rearranged using Microsoft Excel (2017). The statistical analysis was performed using Statistical Package for Social Sciences (SPSS) software (v.21.0). Descriptive and frequency analysis of the number of canals orifices, the shape of canal orifices according to the type of canal present, location of extra canal orifices (if present), the shape of pulp chamber, and the classification of the pulp chamber anatomy of ManFMs and ManSMs according to the Pawar and Singh classification (Pawar & Singh, 2020) (Fig. 1) was done. For assessing the distribution of the data the Kolmogorov-Smirnov Goodness-of-Fit test was applied. To determine the significant difference within each parameter of the study, a chi-square test of proportion was performed. A statistically significant *p* value of <0.05 was considered reliable.

## Results

The current cross-sectional *in-vitro* descriptive study was conducted on extracted permanent ManFMs and ManSMs. The different components of the pulp chamber floor anatomy assessed in our study were the presence of the total number of canal orifices, the shape of the canal orifices according to the type of canal present, location of extra canal orifices (if present), the shape of the pulp chamber, and the classification of the pulp chamber anatomy according to Pawar and Singh classification. The Kolmogorov-Smirnov Goodness-of-Fit test was employed to determine data distribution normality. The *p* value for all parameters were found to be statistically significant (*p* < 0.05), implying that the data were not distributed normally. As a consequence, the Chi square test was applied to assess the difference in proportion.

### Number of canal orifices

**Figure 2:**
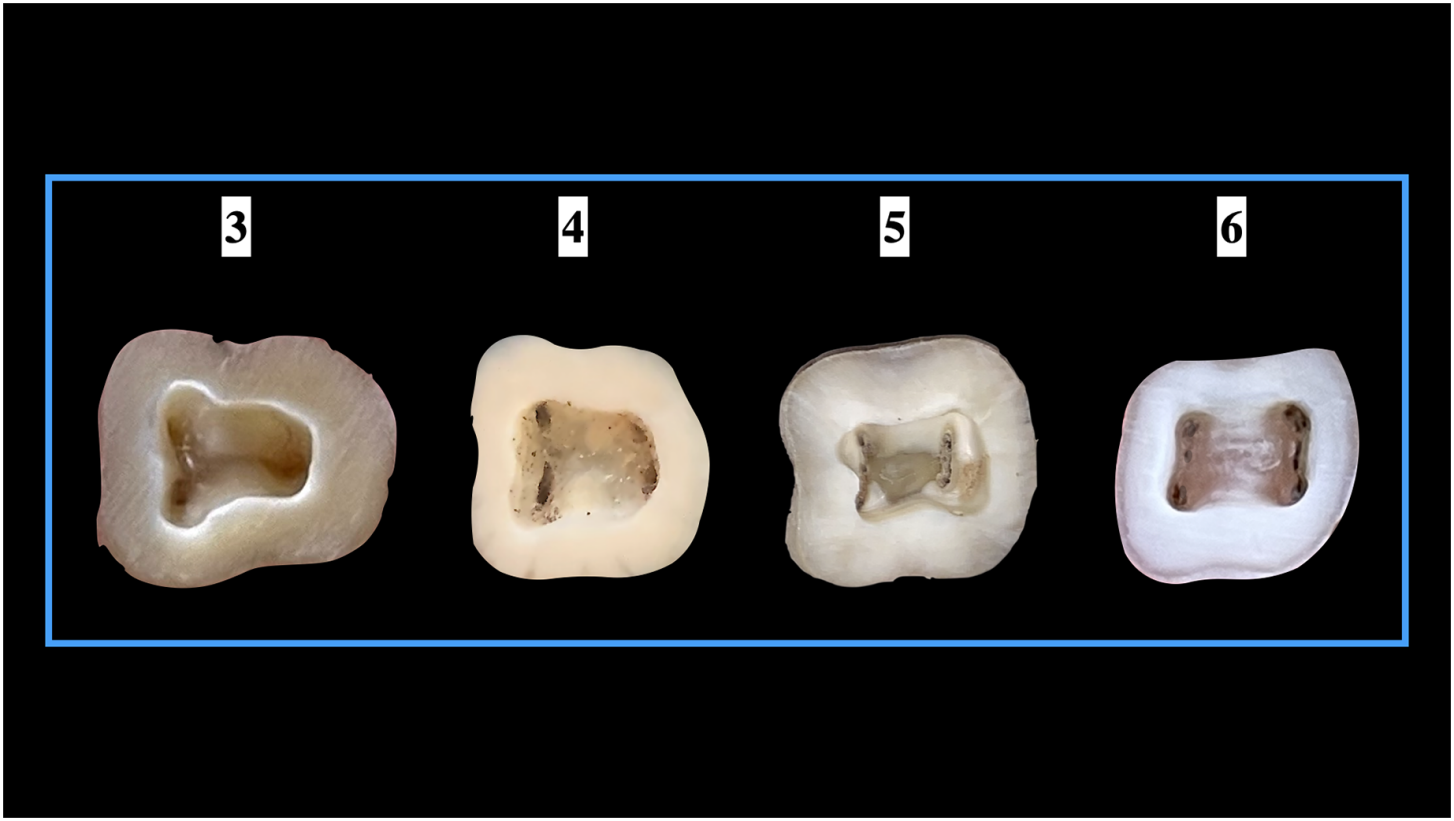
Number of root canal orifices seen in mandibular first molars.

**Figure 3:**
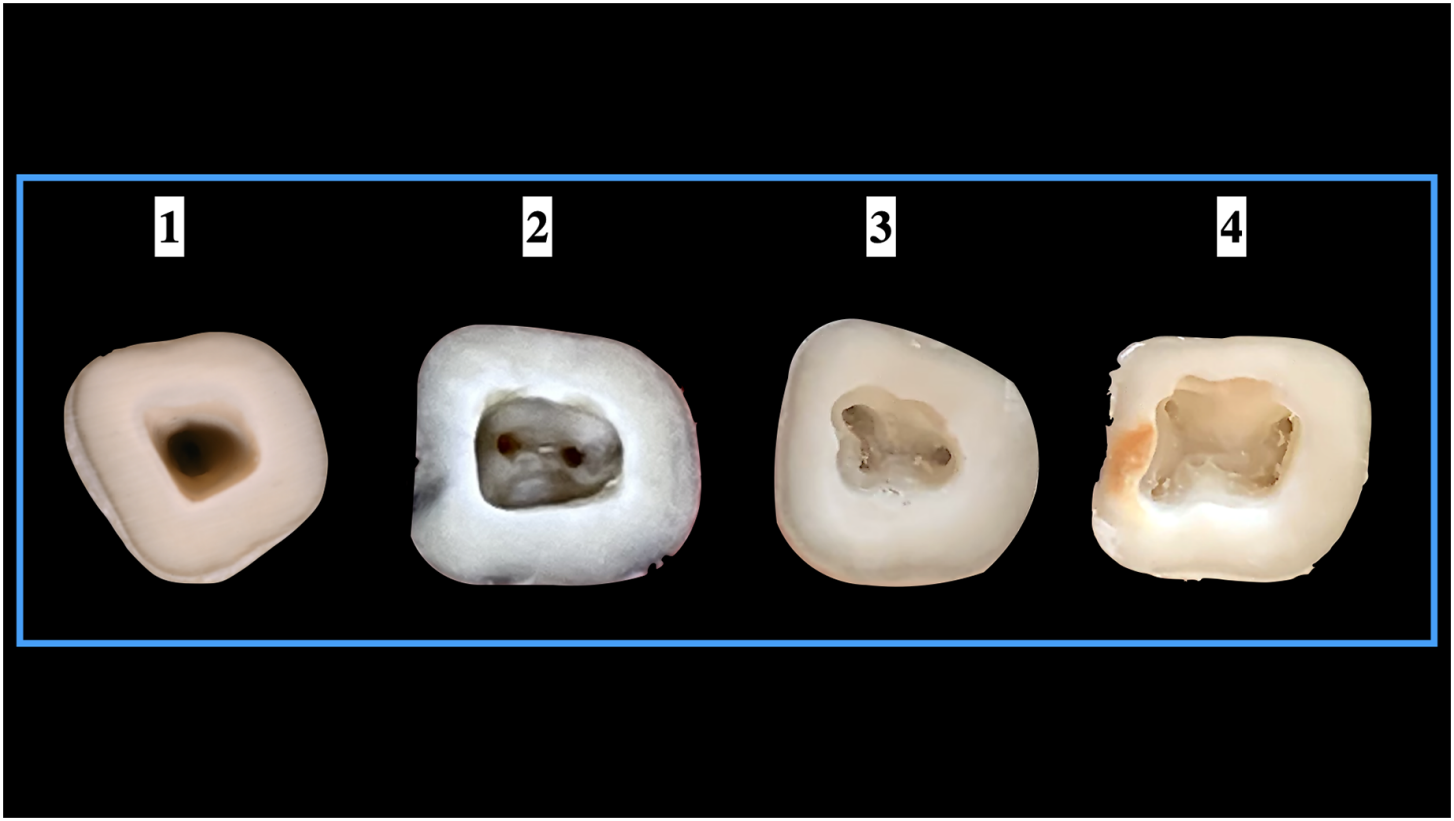
Number of root canal orifices seen in mandibular second molars.

In ManFMs, three root canal orifices were found in 57.81%, followed by four canal orifices in 31.12% specimens, five canals in 8.81% specimens and six canals in 2.15% specimens. In ManSMs, single root canal orifice was observed in 6.53% specimens, two root canal orifices were found in 37.39% specimens, three root canal orifices were found in 39.83% mandibular specimens, four canal orifices in 16.31% specimens. For ManFMs, single or two root canal orifices and for ManSMs, five and six root canal orifices were not observed in any specimens. The findings were significant statistically with three root canal orifices more commonly exhibited by both mandibular molars (Figs. 2 and 3) (Table 1).

**Table 1:** Frequency of the number of canal orifices seen in mandibular first and second molars.

Number of canal orifices	**1**	**2**	**3**	**4**	**5**	**6**	df	Chi square value	*p* value
Mandibular first molars (*n* = 1,067)									
Frequency	–	–	616	334	94	23	4	808.82	0.01*
Percentage	–	–	57.8	31.3	8.8	2.1			
Mandibular second molars (*n* = 1,067)									
Frequency	69	399	425	164	–	–	4	338.29	0.01*
Percentage	6.4	37.4	39.8	16.4	–	–			

**Notes:**

Test applied: Chi-square test.

* *p* ≤ 0.05 statistically significant.

### Shape of the orifices with the type of canal

**Figure 4:**
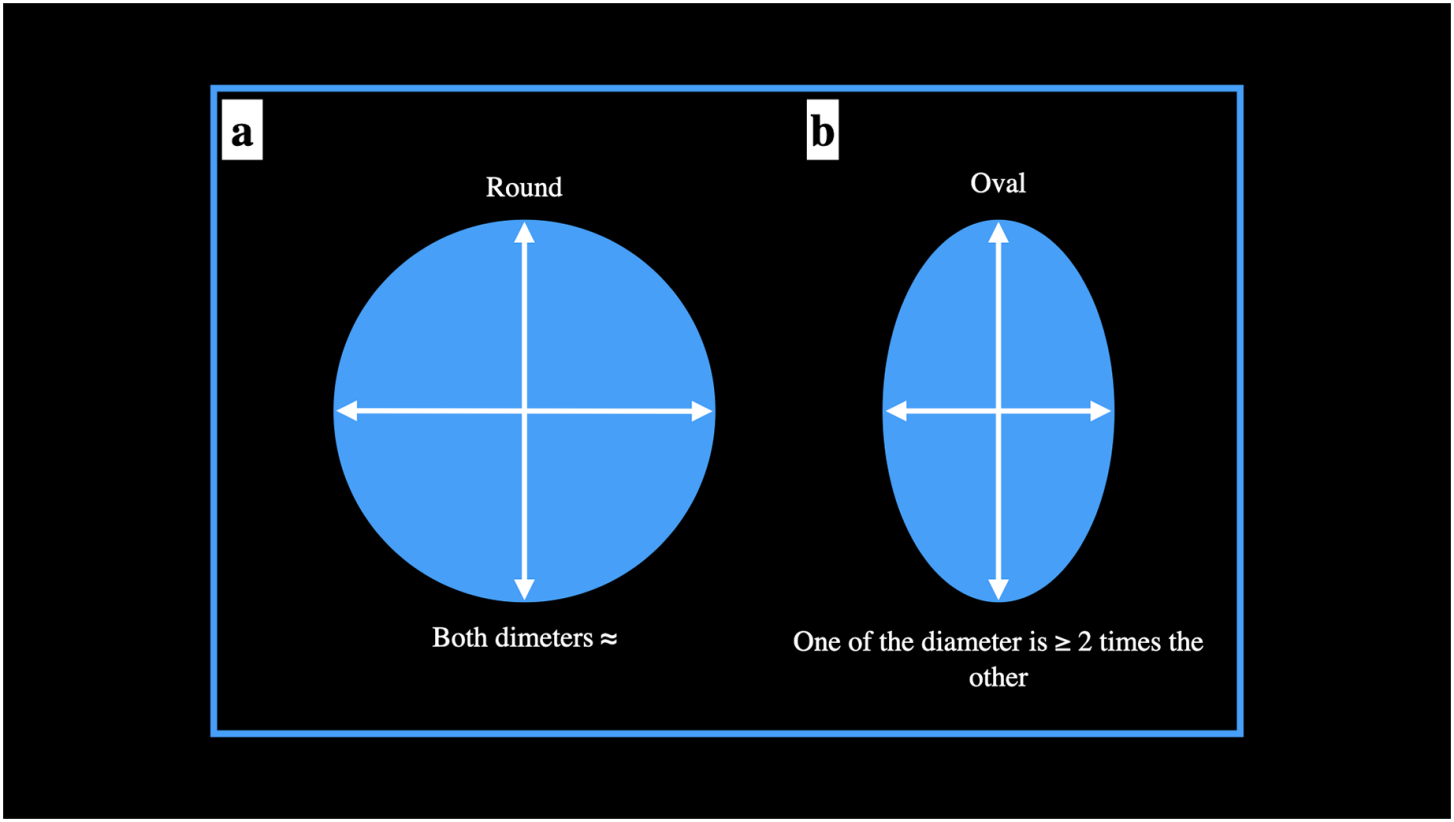
Schematic presentation of the evaluation of the root canal orifice for round and oval shapes.

The shape of the root canal orifice was deemed either oval or round. The orifice was marked as round if one of the diameter was ≈ to the other diameter. The orifice was marked as oval if one of the diameter was $\geq$2 times the other diameter (Jou et al., 2004) (Fig. 4). Any other diameter ratios were labelled as others. For ManFMs, shape of root canal orifice of mesio-buccal and mesio-lingual root canal was round in 55.48% while shape of root canal orifice was oval in 44.52% mandibular specimens. Shape of root canal orifice of disto-buccal and disto-lingual root canal was round in 57.87% mandibular specimens while shape of root canal orifice was oval in 42.13%. Shape of root canal orifice of single distal root canal was round in 11.69% mandibular specimens while shape of root canal orifice was oval in 84.25% and the orifices were of non-classified shape (others) in 4.06% mandibular specimens. The difference was significant statistically (Table 2).

**Table 2:** Frequency of the shapes of canal orifices in Mandibular first molars.

	Mandibular first molar (*n* = 1,067)		
	Mesial	Distal	
Orifice shape	Mesio-buccal and Mesio-lingual	Disto-buccal and Disto-lingual	Distal
Round	55.4%	57.8%	11.7%
Oval	44.6%	42.2%	84.2%
Others	–	–	4.1%
df	2	2	3
Chi square value	12.82	292.91	727.08
*p* value	0.01*	0.01*	0.01*

**Notes:**

Test applied: Chi-square test.

* p ≤ 0.05 statistically significant.

**Figure 5:**
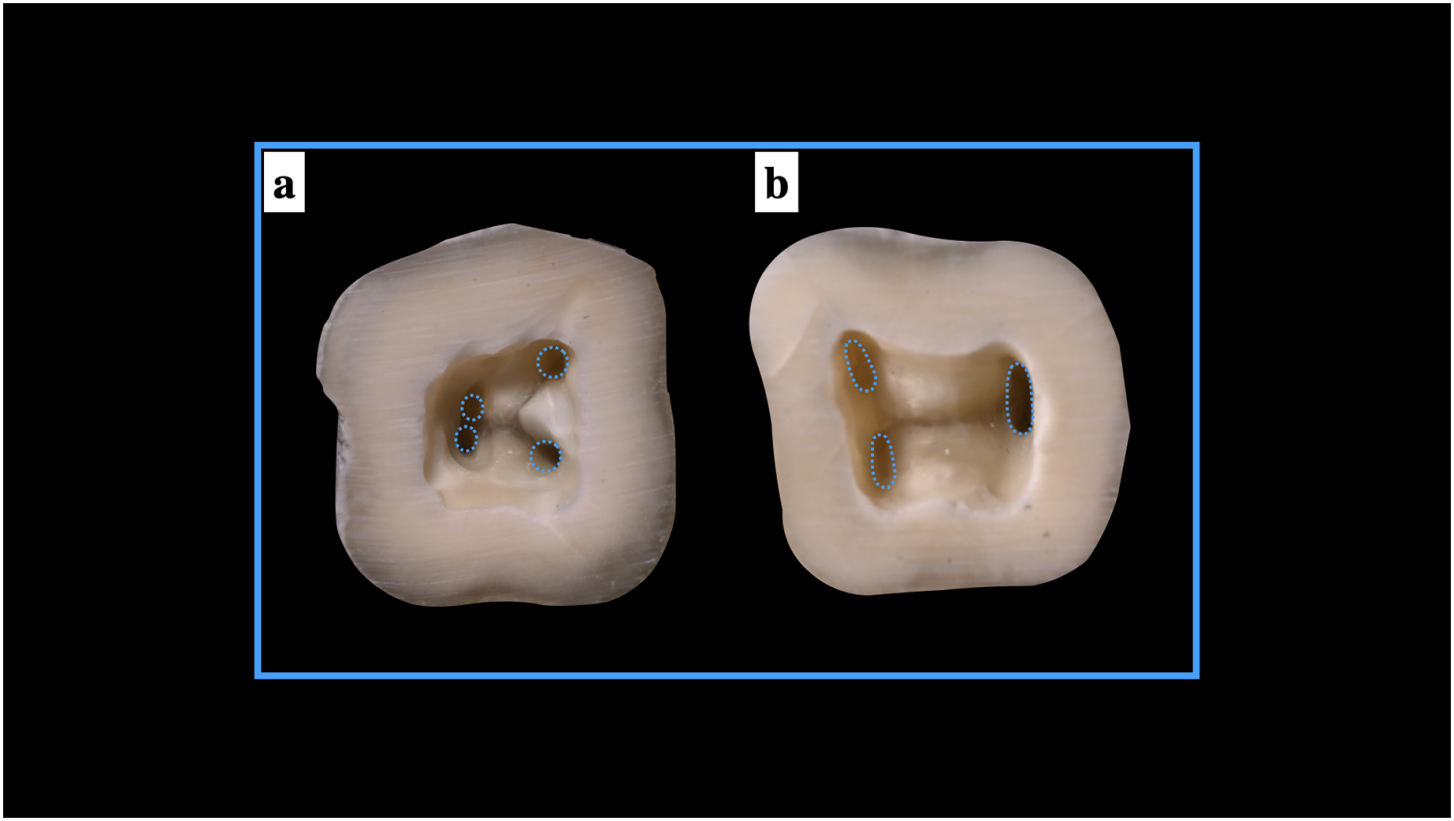
Shapes of the root canal orifices (A) round root canal orifices, (B) oval root canal orifices.

In ManSMs, among the 69 single root canal orifice, 66.66% were round in shape whereas, and 33.34% exhibited a C–shaped orifice. In 399 specimen which exhibited two root canal orifices (mesial and distal), 79.44% and 20.56% of the mesial roots exhibited oval and round shape, respectively. In 599 specimen that exhibited two canal orifices mesially (mesio-buccal and mesio-lingual), the shapes of the orifices were round in 69.61% and oval in 30.39%. For the 824 single distal canal orifice, 74.16% of specimens exhibited oval and 25.84% specimens were found to have round shaped orifice. Of the 174 teeth that possessed two root canal orifices distally (disto-buccal and disto-lingual), 56.89% and 43.11% exhibited round and oval shapes, respectively (Table 3). The representative images of different shapes of root canal orifices in presented in Fig. 5.

**Table 3:** Frequency of the shapes of canal orifices in Mandibular second molars.

	Mandibular second molar (*n* = 1,067)				
	Single orifice (*n* = 69)	Mesial		Distal	
Orifice shape		Single (*n* = 399)	Mesio-buccal and Mesio-lingual (*n* = 599)	Single (*n* = 824)	Disto-buccal and Disto-lingual (*n* = 174)
Round	66.7%	20.6%	69.7%	25.9%	56.9%
Oval	–	79.4%	30.3%	74.1%	43.1%
C-shaped	33.3%	–	**-**	–	–
Others	–	–	–	–	–
df	2	2	2	2	2
Chi square value	740.82	489.05	131.90	274.15	118.49
*p* value	0.01*	0.01*	0.01*	0.01*	0.01*

**Notes:**

Test applied: Chi-square test.

* *p* ≤ 0.05 statistically significant.

### Location of extra canal orifices

Extra root canal orifices were seen in 13.12% of specimen. Location of extra root canal orifice was seen mesially in 83.57% and distally in 16.43% of ManFMs. The difference was significant statistically (*p* < 0.001). There were no extra canal orifices located with respect to the ManSMs.

### Shape of the pulp access cavity

**Figure 6:**
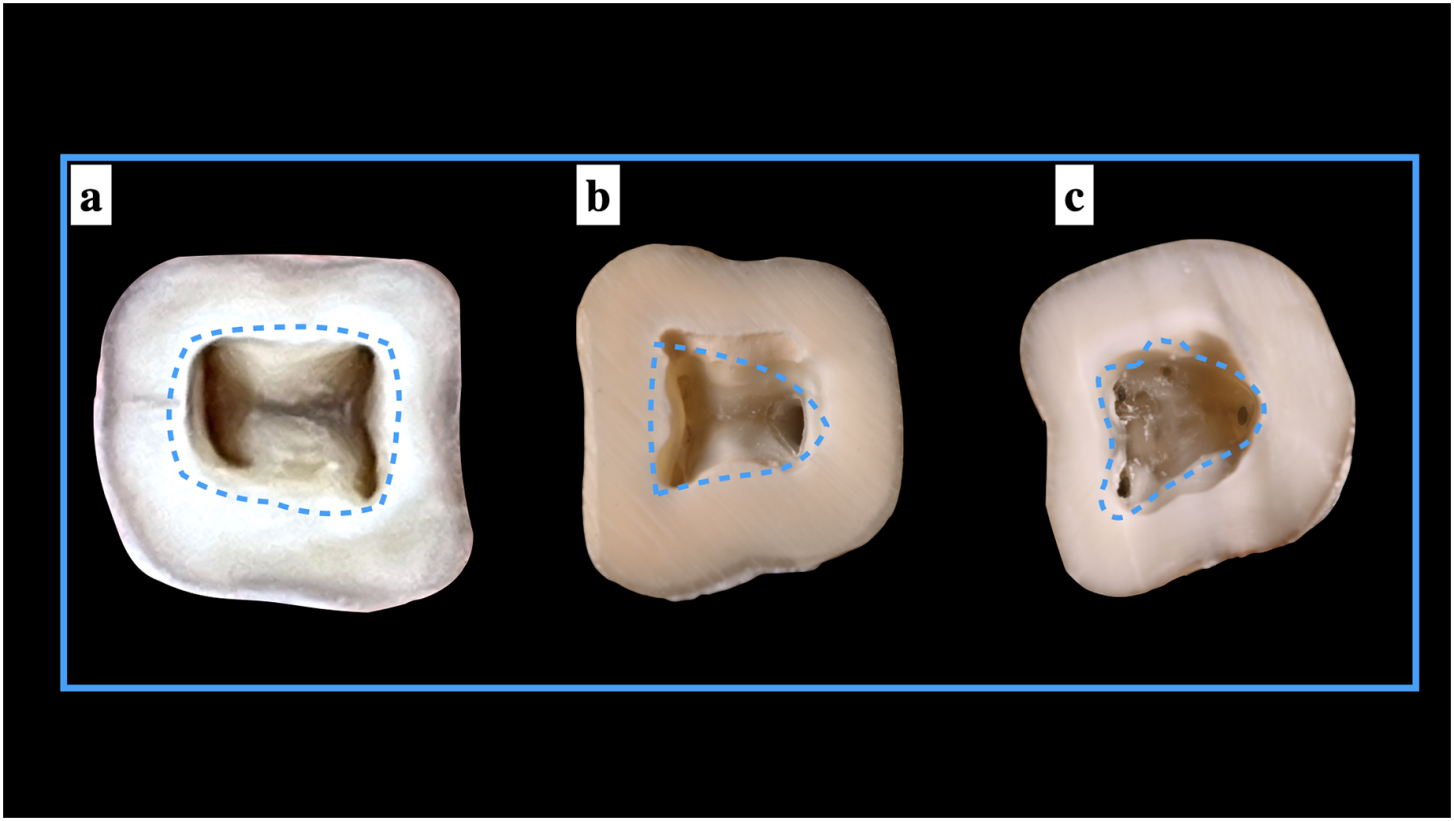
Shape of the pulp access cavities.

Our study observed the shape of the access cavity for ManFMs and MandSMs. About 46.67% and 16.31% of the ManFMs and ManSMs respectively, showed a rectangular shape. A triangular shaped access cavity was observed in 53.33% in ManFMs and 39.83% in ManSMs. Non-classified shape (others) were seen in 43.86% of ManSMs. This difference was significant statistically for only second molars (Table 4). The representative images of different shapes of pulp access cavities in presented in Fig. 6.

**Table 4:** Frequency of ManFMs and ManSMs according to the shape of the pulp access cavity.

	Shape of the pulp access cavity	Frequency (*n* = 1,067)	Percentage (%)	df	Chi square value	*p* value
Mandibular first molar	Rectangle	498	46.7	2	4.72	0.095
	Triangle	569	53.3			
	Others	–	–			
Mandibular second molar	Rectangle	174	16.4	3	141.78	0.001*
	Triangle	425	39.8			
	Others	468	43.8			

**Notes:**

Test applied: Chi-square test.

* *p* ≤ 0.05 statistically significant.

### Implementation of Pawar and Singh classification

**Figure 7:**
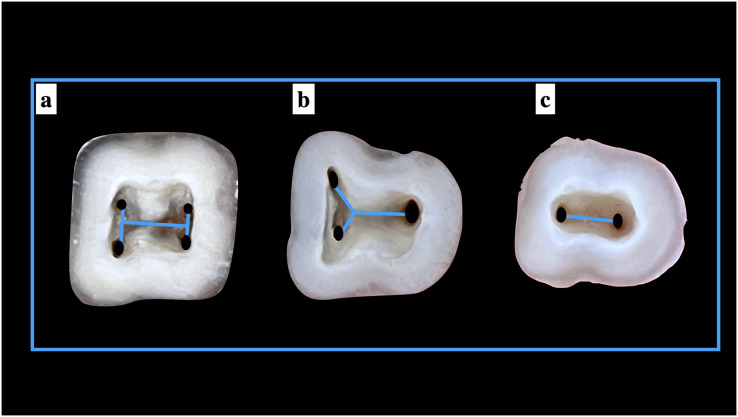
Shape of the root canal orifices anatomy according to Pawar and Singh classification (A) “H” type, (B) “Y” type, and (C) “I” type.

**Figure 8:**
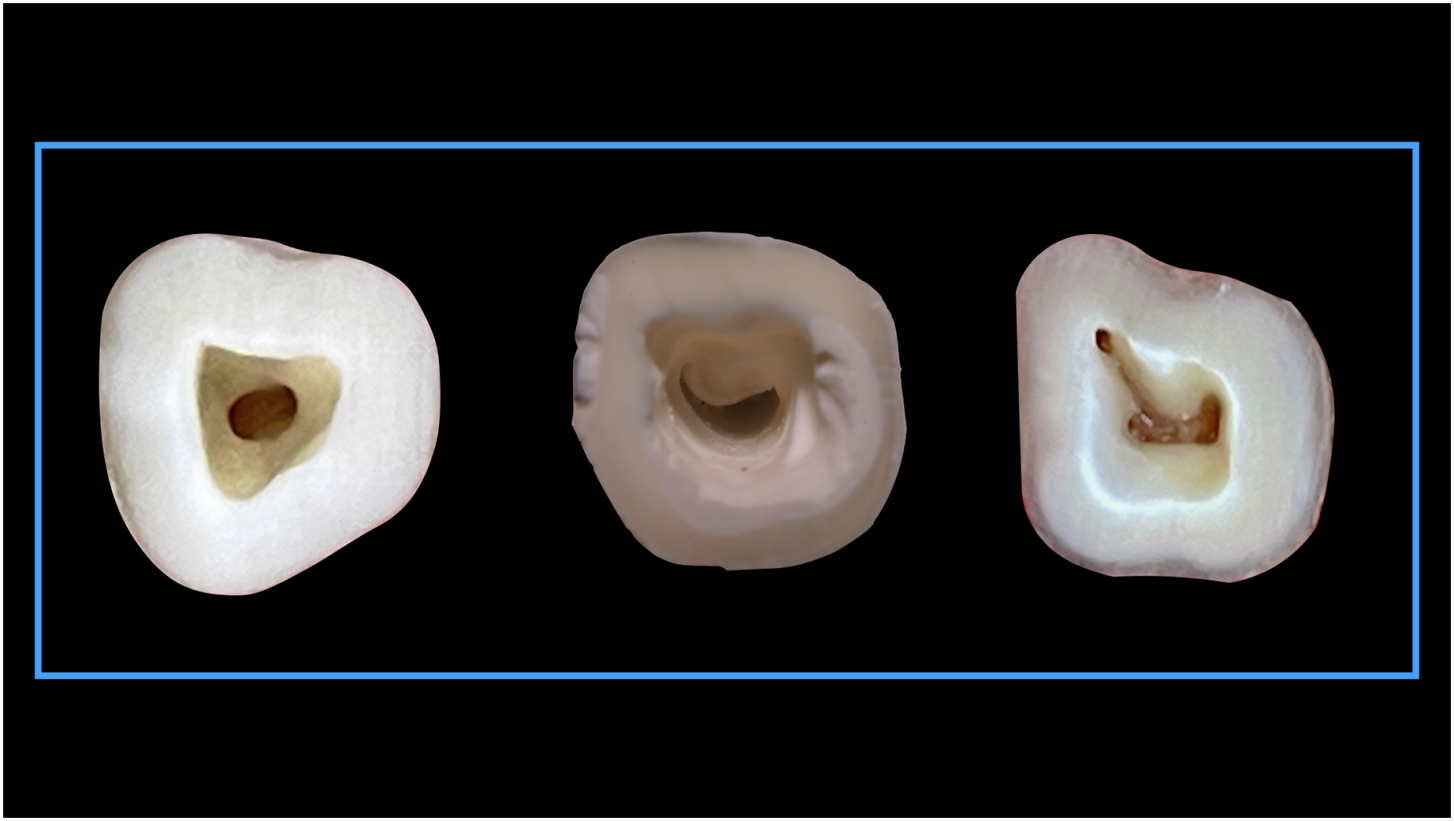
Shape of the root canal orifices anatomy according to Pawar and Singh classification “Others” type.

When the Pawar and Singh classification for pulp chamber floor anatomical features was used, it was discovered that the form of the pulp chamber was “Y” in 57.73% and “H” in 32.27% ManFMs. For the ManSMs, the form of the pulp chamber was determined to be “Y” in 39.83%, “H” in 16.31%, “I” in 37.39%, and “non-classified shape (others)” in 6.47%. The results were statistically significant (Table 5). The application of the classification for ManFMs and ManSMs is represented in Figs. 7 and 8, respectively.

**Table 5:** Frequency of ManFMs and ManSMs according to the classification of orifice in pulp chamber (Pawar and Singh classification).

	Classification of orifice in pulp chamber	Frequency (*n* = 1,067)	Percentage (%)	df	Chi square value	*p* value
Mandibular first molar	H shaped	451	42.2	2	125.51	0.001*
	Y shaped	616	57.8			
	I shaped	–	–			
	Others	–	–			
Mandibular second molar	H shaped	174	16.3	4	338.29	0.001*
	Y shaped	425	39.9			
	I shaped	399	37.4			
	Others	69	6.4			

**Notes:**

Test applied: Chi-square test.

* *p* ≤ 0.05 statistically significant.

## Discussion

The review of literature is suggestive that one of the leading causes of root canal therapy failure is ‘missed canals’ (Tabassum & Khan, 2016; Mustafa et al., 2021). The anatomy and morphology of the root canal system play a vital role in endodontic therapies (Pamukcu Guven, 2020). The most common causes of endodontic treatment failure are missing canals and improper instrumentation (Siqueira, 2001; Iqbal, 2016). Using Pawar and Singh classification (Pawar & Singh, 2020), this article briefly covers the pulp chamber floor architecture of mandibular first and second molars. The shapes of various canal orifices and their role in shaping the form of the pulp chamber floor were evaluated in our study.

The clinician cannot properly identify the number of root canals existing before to initiating therapy. The average number of canals in different teeth is described generically. While the average number of canals in a tooth is useful, it might vary across individuals and between teeth. The existing data on the location of canal orifices has been provided in an unorganised format. Typically, it has been proposed that access to the cavity be achieved in an approximate place in the clinical crown, followed by a search for orifices in the hopes of discovering them. Finding canal orifices in teeth that have been extensively restored, broken down owing to significant caries, or have had incorrect previous access cavities is particularly difficult, since normal anatomy is sometimes severely compromised (Pawar & Singh, 2021).

The anatomy of the pulp chamber floor and walls can be used to determine what morphology is present. In a study of 500 pulp chambers, Krasner and Rankow discovered that the cemento-enamel junction was the most relevant anatomic marker for establishing the location of pulp chambers and root canal orifices (Krasner & Rankow, 2004). They proved the existence of distinct and consistent pulp chamber floor and wall architecture and offered rules to aid clinicians in identifying canal morphology. These laws’ correlations are very useful in detecting calcified canal orifices (McCabe & Dummer, 2012).

Empirical evidence on the root canal morphology of mandibular permanent and primary molars have only evaluated the root canal configurations (de Pablo et al., 2010; Ozcan et al., 2016; Martins et al., 2019); none have adequately evaluated the pulp chamber floor architecture. The mandibular first and second molars are likely the most often affected by caries and frequently need root canal treatment (Umanah, Osagbemiro & Arigbede, 2012). The have two roots (mesial and distal) in their most common form; in rare cases, may have three roots. A two-rooted molar’s canal pattern is normally three canals; two canals orifices in the mesial root and one orifice in the distal root. Because of the presence of two canal orifices mesially, they often have a more intricate root canal system, whereas the distal side has one large canal, however two canal orifices are probable.

If they are thought to have many canals, understanding the coronal pulp chamber floor of these teeth is absolutely essential. Missing root canals are more likely if the practitioner is unfamiliar with the chamber floor. Variation in mandibular molar teeth is caused by a variety of reasons (Kokate, Pawar & Hegde, 2013). Tooth pulp morphology is frequently quite complicated and varied. Few research studies on mandibular molar root canal morphology anticipate that Vertucci’s type IV and type I are the most common configurations in the mesial and distal root of the ManFMs and ManSMs (Gulabivala et al., 2002; Madani et al., 2017; Pawar et al., 2017). Thus, depending on the presence of a root canal system, the morphology of the pulp chamber floor would alter, including the thickness of the thinnest area around the canals throughout endodontic treatments. Accordingly, our study examined the changes that can occur in the pulp chamber floor of molars, which can be determined during the first and most important phase of root canal treatment coronal preparation (Madani et al., 2017).

In the current study, the mandibular first molar exhibited three root canal orifices in 57.73% mandibular specimens, four orifices in 31.31% specimens, five orifices in 8.81% specimens and six canal orifices in 2.15% specimens. No single and two root canal orifices were observed. In mandibular second molar, single root canal orifice was observed in 6.47% specimens, two orifices were found in 37.39% specimens, three orifices were found in 39.83% mandibular specimens, and four canal orifices in 16.31% specimens. No five and six root canal orifices were observed in any specimen of mandibular second molars. Thus, before and during root canal treatment, clinicians should be aware of the complicated root canal morphology of mandibular molars in the Indian population. So, with such intricate root canal designs, analysing the form of the pulp chamber floor before to treatment would result in a favourable outcome with a lower likelihood of failure (Pawar & Singh, 2022).

According to a CBCT investigation by Saberi et al. (2014), 49.5% of ManFMs mesial roots exhibited type IV and 46.5% were type II, suggestive of two canal orifices seen in 96%. The incidence of type IV was 93.9% and type II in 1.7% of the ManFMs investigated in a study by Wang et al. (2010), suggesting of two canal orifices seen in 95.6% of the mesial roots. According to Al-Nazhan (1999), type II (two canal orifices) was the most prevalent type in mesial roots of ManFMs. In our study the occurrence of two mesial canal orifices in ManFMs was almost 89.03%. In our research, we found three orifices mesially in 10.97% of the specimens. The reported prevalence of three root canal orifices mesially of ManFMs ranges from 0.2% to 37.5% (Vertucci, 1984; Shahi et al., 2008; Huang et al., 2010; Wang et al., 2010; Azim, Deutsch & Solomon, 2015). The number of orifices seen in the studied population for mandibular first molars were similar to those seen in Chinese (Gu et al., 2010), Jordinain (Al-Qudah & Awawdeh, 2009), Brazillian (Marceliano-Alves et al., 2019), Iranian (Madani et al., 2017), Spanish (Pérez-Heredia et al., 2017), Sudanese (Ahmed et al., 2007), Burmese (Gulabivala et al., 2001), Ugandan (Rwenyonyi et al., 2007), and Sri Lankan (Peiris et al., 2007). For the mandibular second molars the orifices seen in the current study were in accordance with those exhibited by Korean (Kim, Kim & Kim, 2016), Venezuelan (Gomez, Brea & Gomez-Sosa, 2021), Ugandan (Rwenyonyi et al., 2007), Iranian (Donyavi et al., 2019), Chinese (Zhang et al., 2011), Greek (Kantilieraki et al., 2019), and Spanish (Pérez-Heredia et al., 2017). However the results were contrasting for mandibular second molars with respect to Belgian and Chilean population (Torres et al., 2015).

The shape of the root canal orifice of the mesio-buccal and mesio-lingual root canal orifices in ManFMs was round in 55.48% of the specimens, while it was oval in 44.52% of the specimens. Round form was detected in 69.91% of the mesial roots of ManSMs, whereas oval shape was seen in 30.39%. When two orifices are displayed by ManFMs and ManSMs, this finding emphasises a symmetric relationship in the forms of the root canal orifices.

Our investigation emphasizes on the significance of recognizing the pulp chamber floor morphology of mandibular molars, as well as the patterns that a dentist may confront during endodontic treatment. We have focussed on the complex changes in root canal networks that result in changes in the morphology of the pulp chamber floor. These disparities in the shape of the floor are mostly influenced by the overall number of canals housed, the variable shape of the canal orifices, and their integration, which culminates in a distinct shape.

The focus of this study was to explore the architectural style of the coronal pulp chamber floor to see whether there are any unique, reproducible characteristics or patterns that can be defined. If these markers are present, the quest for orifices might become more systematic and accurate. Furthermore, it is true that a well-designed access cavity is anticipated for a satisfactory endodontic consequence (Pawar & Singh, 2021). Most root canal treatment failures are caused by the inability to locate additional canals, debride them, and fill them. A clinician’s understanding and knowledge of pulp space anatomy and its frequent anatomic variation contributes in the attainment of an acceptable endodontic outcome (Cantatore, Beryttie & Castellucci, 2006). A perforation in the pulp chamber floor, inadequate de-roofing, iatrogenic pulp exposure during cavity preparation, dentinal gouging, and missing canals during the endodontic treatment are all possible complications. All of this is preventable if a broad understanding of the anatomy of the pulp chamber floor is sustained.

The current descriptive study focused primarily on the number of root canal orifices that are usually discovered during endodontic treatment of mandibular first and second molars. The orifice morphologies of these teeth have also been investigated, enabling the clinician to foresee the root canal’s shape and choose an instrumentation strategy appropriately. The main advantage of the utilised classification may be that it would facilitate communication between the specialist and the general practitioner and direct them to alter the access cavity. Also a large sample size and simple photographic examinations with varied magnification may be considered benefits.

There are still some obvious limitations to this study, though, which can be taken into consideration for future investigation. The distribution of root canal orifices, which is frequently seen in patients’ teeth from various age groups, is one of the drawbacks that would need to be accounted for. The relationship between the number of orifices seen and the number of roots present, the number of root canals to full working length, and partial calcification of the root canals in a single tooth might also be explored.

## Conclusion

Since it is impossible to quantify how many root canals are in a tooth before treatment is commenced, only comprehensive overview of the PCF anatomy may provide improved precision regarding the overall number of root canals in a particular tooth. Our study has included complete description of the distinguishing features of the ManFMs and ManSMs. This knowledge may aid in clinically informed chair-side decision making, which may serve as a foundation before delving into the complicated root canal morphology. More thorough research investigations may be undertaken to compare the racial and ethnic propensity of root canal system anatomical patterns. The establishment of the alphabetical Pawar Singh Molar pulp chamber floor classification and its application to the identification of the placement of root-canal orifices may be credited with the foundational anatomical concepts that serve as the basis for endodontic practise. Practitioners can use these patterns to hunt for unrecognised canal orifices if they can identify some of the canals that form a distinctive alphabet in any tooth by their specific orifice location on the pulp chamber floor.

## Supplemental Information

10.7717/peerj.14392/supp-1 Supplemental Information 1 Raw Data.

## Additional Information and Declarations

### Data Availability

The following information was supplied regarding data availability: The raw data is available in the Supplemental Files.
